# Relationship between Habitual Intake of Vitamins and New-Onset Prediabetes/Diabetes after Acute Pancreatitis

**DOI:** 10.3390/nu14071480

**Published:** 2022-04-01

**Authors:** Claire F. Norbitt, Wandia Kimita, Sakina H. Bharmal, Juyeon Ko, Maxim S. Petrov

**Affiliations:** School of Medicine, University of Auckland, Auckland 1023, New Zealand; cnor327@aucklanduni.ac.nz (C.F.N.); wandia.kimita@auckland.ac.nz (W.K.); s.bharmal@auckland.ac.nz (S.H.B.); ju.ko@auckland.ac.nz (J.K.)

**Keywords:** carotenoids, vitamin B3, habitual vitamin intake, acute pancreatitis, prediabetes, diabetes, glucose metabolism, insulin traits

## Abstract

Vitamins have many established roles in human health. However, the role of habitual dietary intake of vitamins in glucose homeostasis in individuals after acute pancreatitis (AP) is yet to be elucidated. The aim was to investigate the associations between habitual intake of fat- and water-soluble vitamins/vitamers and markers of glucose metabolism (fasting plasma glucose (FPG), homeostasis model assessment insulin resistance (HOMA-IR) index, and homeostasis model assessment β-cell function (HOMA-β)) in individuals after AP. A total of 106 participants after AP were included in this cross-sectional study and were grouped based on glycaemic status: new-onset prediabetes/diabetes after AP (NODAP), pre-existing prediabetes/type 2 diabetes (T2DM), and normoglycaemia after AP (NAP). Habitual intake of seven fat-soluble vitamins/vitamers and seven water-soluble vitamins were determined by the EPIC-Norfolk food frequency questionnaire. Multiple linear regression analyses were conducted using five statistical models built to adjust for covariates (age, sex, daily energy intake, visceral/subcutaneous fat volume ratio, smoking status, daily alcohol intake, aetiology of AP, number of AP episodes, cholecystectomy, and use of antidiabetic medications). In the NODAP group, three fat-soluble vitamins/vitamers (α-carotene, β-carotene, and total carotene) were significantly associated with HOMA-β. One water-soluble vitamin (vitamin B3) was also significantly associated with HOMA-β in the NODAP group. None of the studied vitamins were significantly associated with FPG or HOMA-IR in the NODAP group. Prospective longitudinal studies and randomised controlled trials are now warranted to investigate if the observed associations between vitamin/vitamer intake and NODAP are causal and to unveil the specific mechanisms underlying their involvement with NODAP.

## 1. Introduction

Vitamins and vitamers are essential micronutrients required to maintain metabolic health [1,2]. There are 13 known vitamins and several vitamers required in small quantities for metabolic health (e.g., energy metabolism, antioxidant function, and enzymatic functions) [2,3,4,5,6,7,8]. There are two subtypes of vitamins classified by their solubility—fat-soluble and water-soluble vitamins. Most vitamins (such as vitamins A, C, E, and K, majority of B complex vitamins) are not endogenously synthesised by the body and are predominantly obtained through the diet [5]. The structural differences between the groups of vitamins influence their method of absorption [3]. Low intake of vitamin-rich foods or reduced vitamin absorption can cause oxidative stress-driven disorders such as insulin resistance [9,10], β-cell dysfunction [11], and metabolic syndrome [8,12]. Reduced fat- and water-soluble vitamin intake is also associated with type 2 diabetes. For instance, a large prospective cohort study showed that low intake of fat-soluble vitamins E and K increased risk of this type of diabetes [13]. Other studies demonstrated that low intake of water-soluble vitamins C, B2, and B9 was associated with increased risk of type 2 diabetes [14,15].

Another example of a disease marked by oxidative stress is acute pancreatitis (AP). Damage to pancreatic cells during an attack of AP can cause subsequent endocrine and exocrine dysfunctions [16,17]. A systematic literature review and meta-analysis by the COSMOS group showed that exocrine pancreatic dysfunction occurs in 29% of patients following an episode of AP [18]. Exocrine pancreatic insufficiency impairs digestion and absorption of food and nutrients, resulting in deficiency of vitamins [19]. A large cohort study conducted by the COSMOS group demonstrated that individuals after AP with exocrine pancreatic insufficiency had a significantly increased risk of new-onset diabetes after AP, irrespective of the disease severity [20]. New-onset prediabetes/diabetes after AP (NODAP) develops in about 40% of individuals after an episode of AP and individuals with NODAP are characterised by chronic low-grade inflammation, impaired lipid metabolism, iron metabolism, and islet dysfunction [17,21,22]. NODAP is a clinically distinct entity to type 2 diabetes, yet often misclassified and misdiagnosed as type 2 diabetes [21,23,24]. Individuals with NODAP have worse glycaemic control [21], increased need for insulin therapy [21], higher incidence of pancreatic cancer [25], and higher hospitalisations and mortality than individuals with type 2 diabetes [26]. Nonetheless, there are no specific guidelines for optimal nutrition therapy for new-onset diabetes after AP, with patients typically receiving generalised type 2 diabetes nutrition advice focusing on macronutrient distribution, portion sizes, and intake of minimally processed foods [27,28]. Emerging evidence suggests a role of micronutrients, particularly mineral intake, in the development of new-onset diabetes after AP [29,30]. To date, intake of other micronutrients (specifically vitamin intake) has never been investigated in the context of new-onset diabetes after AP.

Therefore, this study aimed to investigate the relationships between habitual vitamin intake and fasting plasma glucose (FPG), homeostasis model assessment insulin resistance (HOMA-IR) index, and homeostasis model assessment β-cell function (HOMA-β) index in individuals after AP.

## 2. Materials and Methods

### 2.1. Study Design

This study was a cross-sectional investigation of individuals after an episode of AP. This study was part of the ANDROMEDA (Assessment of Nutritional and DietaRy factOrs in Metabolic Disorders after pAncreatitis) project conducted by the COSMOS group. Ethical approval for this study was granted by the Health and Disability Ethics committee (13/STH/182). Individuals were eligible to participate if they had a primary diagnosis of AP (defined by international guidelines [31]), were aged 18 years or older, resided in Auckland (New Zealand) throughout the study, and gave informed consent for participation. Individuals were not eligible to participate in the study if they had history of type 1 or gestational diabetes, a diagnosis of chronic pancreatitis, intra-operative diagnosis of pancreatitis, post-endoscopic retrograde cholangiopancreatography pancreatitis, pregnancy throughout or after AP attack, history of steroid use, malignancy, coeliac disease, or cystic fibrosis.

### 2.2. Study Groups

According to the ‘DEP criteria’, individuals were categorised into three non-overlapping groups based on HbA1c and FPG [32].

Normoglycaemia after AP (NAP) group: individuals who had HbA1c < 5.7% (39 mmol/mol) and/or FPG < 100 mg/dL (5.6 mmol/L) at the time of their primary attack of AP and at the time of the study were deemed to have NAP.

Type 2 diabetes or prediabetes (T2DM) group: individuals who had an HbA1c ≥ 5.7% (39 mmol/mol) and/or FPG ≥ 100 mg/dL (5.6 mmol/L) at the time of their primary attack of AP and at the time of their participation in the study were deemed to have T2DM.

New-onset diabetes or prediabetes after AP (NODAP) group: individuals who were normoglycaemic prior to and throughout their primary attack of AP and had HbA1c ≥ 5.7% (39 mmol/mol) and/or FPG ≥ 100 mg/dL (5.6 mmol/L) at follow-up were deemed to have NODAP. Participants who had FPG > 100 mg/dL (5.6 mmol/L) but HbA1c < 5.7% (39 mmol/mol) during their primary attack of AP were not considered in this study to account for effects of transient stress hyperglycaemia [17].

### 2.3. Ascertainment of Vitamin Intake

The EPIC-Norfolk food frequency questionnaire (FFQ) was used to collect habitual dietary intake data of study participants in the 12 months prior to recruitment [33]. The extensively validated, semi-quantitative, and self-administered FFQ assesses the frequency of intake of 130 consumed foods. Additionally, information on types and brands of commonly consumed foods was collected (cereal, milk, meat, and cooking oils). Data gathered from the FFQ were then analysed using the FETA (FFQ EPIC Tool for Analysis) software (V2.53, University of Cambridge, Cambridge, UK) to ascertain daily intake of seven fat-soluble vitamins and vitamers (α-carotene (µg), β-carotene (µg), retinol (µg), total carotene (µg) vitamin A/total retinol equivalents (µg), vitamin D (µg), and vitamin E (mg)), and seven water-soluble vitamins (vitamin B1 (mg), vitamin B2 (mg), vitamin B3 (mg), vitamin B6 (mg), vitamin B9 (µg), vitamin B12 (µg), vitamin C (mg)). Habitual vitamin intake was measured from dietary intake only and intake from vitamin supplementation was excluded from this study. FFQ data were excluded from the study if the FFQ was incomplete (ten or more questions were left unanswered) to limit underestimation of habitual intake [33]. If the ratio of estimated total energy intake (estimated from the FFQ data) and estimated basal metabolic rate (calculated by the Harris-Benedict equation) were more than two standard deviations (SD) outside the mean ratio (i.e., <0.28 and >1.82), FFQ data were excluded [33].

### 2.4. Laboratory Assays

All participants were required to fast 8 h before blood collection by certified phlebotomists. Fresh blood samples were analysed, with HbA1c (mmol/mol), FPG (mmol/L), and fasting insulin (mU/L) measured by LabPlus (International Accreditation New Zealand accredited medical laboratory at Auckland City Hospital). HbA1c was analysed using the boronate affinity chromatography assay (©2015 Roche Products (New Zealand) Ltd., Auckland, New Zealand and Roche Diagnostics NZ Ltd., Auckland, New Zealand). FPG was measured using an enzymatic colourimetric assay (©2015 F. Hoffmann-La Roche Ltd., Basel, Switzerland). Fasting insulin was measured using chemiluminescence sandwich immunoassay (Roche Diagnostics, Auckland, New Zealand). Oxford University’s Homeostasis Model Assessment calculator (HOMA2) was used to estimate HOMA-IR and HOMA-β indices (version 2.2.4 Diabetes Trials Unit, University of Oxford, Oxford, UK).

### 2.5. Covariates

The COSMOS team collected anthropometric and demographic data from participants. Participants underwent magnetic resonance imaging (MRI) to measure abdominal visceral fat volume (VFV) and subcutaneous fat volume (SFV). A 3T MAGNETOM Skyra scanner (Siemens, Erlangen, Germany) was used to conduct MRI scans at the Centre for Advanced Magnetic Resonance Imaging (University of Auckland, Auckland, New Zealand). Axial T1-weighted volumetric interpolated breath-hold examination Dixon sequence was applied, with participants lying supine and holding breath at maximum expiration, as reported elsewhere [34]. Fat-only images between L2 and L5 vertebral lumbar levels were quantified using ImageJ software (National Institutes of Health, Bethesda, MD, USA) [35]. Greyscale pixels of the slice series were converted into binary images using the threshold function of ImageJ, as per the global histogram-derived method [34]. Non-adipose tissue was excluded from the visceral fat measurements. Last, the total number of pixels from the slices series was calculated and multiplied by the pixel area and slice thickness to obtain the VFV and SFV [36]. VFV and SFV were measured by two independent and blinded raters, and the average values of the two measurements were used as the final values of VFV and SFV. The ratio of visceral to subcutaneous fat volume (V/S fat volume ratio) was then calculated. Average daily energy intake (kcal) and alcohol intake (g/day) was obtained using the FFQ and quantified by the FETA software (University of Cambridge, Cambridge, UK) [33]. Tobacco smoking status was recorded as either ‘yes’ or ‘no’ using a standardised questionnaire [37]. Data on antidiabetic medication usage, cholecystectomy, and AP aetiology were obtained from participants’ health records.

### 2.6. Statistical Analyses

Statistical analyses were performed using SPSS 27.0 software (IBM Corporation, Armonk, NY, USA). One-way ANOVA was used to investigate differences in participants’ characteristics between the study groups (NODAP, T2DM, and NAP). Data were presented as mean (standard deviation) or frequency (percentage), and p values were deemed statistically significant if less than 0.05. Associations between the habitual intake of the investigated fat-soluble and water-soluble vitamins and FPG, HOMA-β, and HOMA-IR were examined for each study group. FPG, HOMA-β, and HOMA-IR were the dependent variables and the vitamin variables were the independent variables. All investigated vitamins/vitamers showed skewed distribution (based on the Shapiro-Wilk test) and therefore were logarithmically transformed to account for non-normal distribution. A total of five statistical models were built for multiple linear regression analyses. Model 1 was unadjusted; model 2 was adjusted for age, sex, and daily energy intake; model 3 was adjusted for variables included in model 2 and V/S fat volume ratio; model 4 was adjusted for variables included in model 3 as well as smoking status and daily alcohol intake; model 5 was adjusted for variables included in model 4 and aetiology of AP, number of AP episodes, cholecystectomy, and use of antidiabetic medications. Data were presented as R^2^, unstandardised B, p value, and 95% confidence interval. P values less than 0.05 were deemed statistically significant in all analyses, and data were not corrected for multiple tests.

## 3. Results

### 3.1. Study Cohort

Of the 117 individuals who enrolled in the study, 106 participants were used for analyses. 11 participants were excluded for more than 10 unanswered FFQ questions and estimated basal metabolic exceeding two SDs outside the mean ratio. Of the 106 included participants, 37 participants made up the NODAP group, 37 the T2DM group, and 32 the NAP group. The mean time elapsed after an attack of AP was of 26 months. The study groups did not differ significantly in terms of the intake of all the vitamin/vitamers. Other descriptive characteristics are presented in Table 1.

### 3.2. Fat-Soluble Vitamin Intake and Markers of Glucose Metabolism in the Study Groups

#### 3.2.1. Fasting Plasma Glucose

In the NODAP and T2DM groups, associations between FPG and the investigated fat-soluble vitamins/vitamers were not statistically significant (Table 2).

In the NAP group, FPG was significantly associated with two fat-soluble vitamins/vitamers (retinol and vitamin E). Retinol intake was significantly directly associated with FPG in the most adjusted models (model 4: *p* = 0.046, model 5: *p* = 0.038). Vitamin E intake was significantly inversely associated with FPG in model 2 (*p* = 0.040).

#### 3.2.2. HOMA-β

In the NODAP group, HOMA-β was significantly associated with three fat-soluble vitamins/vitamers (α-carotene, β-carotene, and total carotene). α-carotene was significantly directly associated with HOMA-β in the unadjusted model (model 1: *p* = 0.034) and adjusted models (model 3: *p* = 0.042, model 4: *p* = 0.023, model 5: *p* = 0.013). β-carotene was significantly directly associated with HOMA-β in the most adjusted models (model 4: *p* = 0.044, model 5: *p* = 0.035). Total carotene was significantly directly associated with HOMA-β in the most adjusted models (model 4: *p* = 0.039, model 5: *p* = 0.029).

In the T2DM or NAP groups, HOMA-β was not significantly associated with any of the investigated fat-soluble vitamins/vitamers (Table 3).

#### 3.2.3. HOMA-IR

In the NODAP, T2DM and NAP groups, associations between HOMA-IR and the investigated fat-soluble vitamins/vitamers were not statistically significant (Table S1).

### 3.3. Water-Soluble Vitamin Intake and Markers of Glucose Metabolism in the Study Groups

#### 3.3.1. Fasting Plasma Glucose

In the NODAP group, associations between FPG and the investigated water-soluble vitamins were not statistically significant (Table 4).

In the T2DM group, FPG was significantly associated with three water-soluble vitamins (Vitamin B1, vitamin B2, and vitamin B12) (Table 4). Vitamin B1 was significantly inversely associated with FPG in adjusted model 4 only (*p* = 0.036). Vitamin B2 was significantly inversely associated with FPG in adjusted models (model 2: *p* = 0.030, model 3: *p* = 0.041, model 4: *p* = 0.046). Vitamin B12 was significantly inversely associated with FPG in the unadjusted model (model 1: *p* = 0.001) and adjusted models (model 2: *p* = 0.002, model 3: *p* = 0.003, model 4: *p* = 0.024).

In the NAP group, FPG was significantly associated with one water-soluble vitamin (vitamin B3). Vitamin B3 was significantly inversely associated with FPG in adjusted model 2 (*p* = 0.030).

#### 3.3.2. HOMA-β

In the NODAP group, HOMA-β was significantly associated with one water-soluble vitamin (vitamin B3) (Table 5). Vitamin B3 was significantly directly associated with HOMA-β in the most adjusted models (model 4: *p* = 0.035, model 5: *p* = 0.041).

In the T2DM and NAP groups, associations between HOMA-β and the investigated water-soluble vitamins were not statistically significant.

#### 3.3.3. HOMA-IR

In the NODAP group, T2DM and NAP groups, associations between HOMA-IR and the investigated water-soluble vitamins were not statistically significant Table S2.

## 4. Discussion

The present study was the first to investigate the associations between a comprehensive vitamin profile and markers of glucose metabolism (FPG, HOMA-β, and HOMA-IR) in individuals after an attack of AP. A key finding of this study was that, of the seven fat-soluble vitamins/vitamers, significant direct associations between habitual intake of fat-soluble vitamers α-carotene, β-carotene, and total carotene and HOMA-β were found in the NODAP group. Also, of the seven water-soluble vitamins, a significant direct association was observed between vitamin B3 and HOMA-β in individuals with NODAP.

### 4.1. Fat-Soluble Vitamins

Fat-soluble vitamins (vitamins A, D, E, and K) are hydrophobic compounds and, therefore, are insoluble in the aqueous environment of the gastrointestinal tract [3]. Fat-soluble vitamins are stored in the liver and adipose tissue and, hence, are very slowly excreted from the body. Therefore, very high intakes of fat-soluble vitamins can be detrimental to health [3,38]. Fat-soluble vitamin deficiencies are rare; however, insufficiency may occur in sub-populations including individuals with very low fat intake, low energy intake, or vegetarian/vegan diets [38]. Due to the dependence on dietary fat intake for absorption of fat-soluble vitamins, individuals with malabsorptive conditions, such as exocrine pancreatic dysfunction (EPD), may also develop subsequent deficiencies in these vitamins [19,39,40].

Vitamin A is available in the diet in two forms—retinol (preformed vitamin A) and provitamin carotenoids [41]. There are several forms of carotenoids, with α-carotene and β-carotene being among the most abundant in the human diet and body [41]. Carotenoids are endogenously converted into retinol and contribute to overall vitamin A status. They have potent antioxidant properties that have been found to have a beneficial role in eye health, cognitive function, and the prevention of several diseases including cardiovascular diseases and cancer [42,43]. Dietary carotenoids have also been shown to be associated with the incidence of type 2 diabetes. Quansah et al. observed that increased intake of α-carotene had a 48% and 39% reduction in diabetes risk in Korean men and women, respectively [44]. Additionally, β-carotene intake also reduced the risk of diabetes in men (though no association was found in women) [44]. Another large prospective study showed that higher dietary intake of α-carotene (0.7 mg/day) was associated with a 15% lower risk of type 2 diabetes and β-carotene (3.5 mg/day) was associated with a 22% reduced risk of diabetes, compared with the lowest quartile of the vitamins [45]. A community-based longitudinal study showed that increasing dietary intake of β-carotene by 1.4 mg/day was associated with up to a 34% lower risk of incident diabetes in elderly Swedish men [46]. Men in the highest tertile of β-carotene intake (>1.9 mg/day) at age 70 had up to a 50% lower risk of type 2 diabetes compared with the lowest tertile (<1.0 mg/day) [46]. Additionally, an 0.2 umol/L increase of serum β-carotene at age 50 years was associated with 0.08 units higher insulin sensitivity (determined with the use of euglycaemic-hyperinsulinaemic clamp) in nondiabetic participants at age 70 years. However, insulin secretion was not influenced significantly by serum β-carotene levels [46]. Harari et al. found that serum α-carotene, β-carotene, and total carotenoids were inversely associated with fasting insulin and HOMA-IR in an Australian adult population [47]. Mirmiran et al. also observed that increased dietary intake of β-carotene, but not other carotenoids, was significantly associated with a lower risk of HOMA-IR in Iranian adults [48]. Serum β-carotene concentration was inversely associated with HOMA-IR in middle-aged Japanese individuals [49]. A meta-analysis of prospective observational studies also concluded that dietary intake and circulating concentrations of total carotenoids are associated with beneficial effects on reducing the risk of type 2 diabetes in a population at high risk of type 2 diabetes [49]. In this meta-analysis, β-carotene intake was also consistently inversely associated with diabetes risk.

The present study was the first to investigate the associations between habitual carotenoid intake and insulin traits in individuals after an attack of AP. We found that dietary intake of α-carotene, β-carotene, and total carotene intake was significantly and directly associated with HOMA-β, indicating detrimental effects of deficient carotenoid intake on insulin secretion. Specifically, for every 1% decrease in α-carotene, β-carotene, and total carotene intake, HOMA-β decreased by 0.42%, 0.60%, and 0.63%, respectively. These results indicate that increased intake of α-carotene, β-carotene, and total carotenoids may have beneficial effects on insulin secretion in individuals with NODAP. We found no association with other markers of glucose metabolism (FPG and HOMA-IR). Several intrinsic and extrinsic factors are associated with carotenoid status; therefore, dietary intake may not truly reflect carotenoid status in the body [50]. In our unique cohort of individuals following an attack of AP, there may be mechanistic differences in the absorption or utilisation of fat-soluble vitamins, such as carotenoids, compared with those with type 2 diabetes [51]. It is not uncommon for individuals to develop EPD following an attack of AP, which leads to maldigestion and malabsorption of nutrients, particularly of fat and fat-soluble vitamins [20,52]. It has also previously been established that there is an association between EPD and NODAP, where individuals with EPD have a significantly increased risk of developing NODAP [20]. It was suggested that deficiency in fat-soluble vitamins may have a role in this association as no significant correlation was found between exocrine pancreatic dysfunction and NODAP when individuals were taking fat-soluble vitamin supplements [20]. It is worth noting that pancreatic enzymes and serum carotenoid levels were not measured in the present study. Hence, we were not able to determine if EPD or low serum levels of these vitamins contributed to the observed results [53].

Vitamin D has two primary forms—vitamin D3 or cholecalciferol (which is endogenously synthesised in the skin after exposure to ultraviolet light) and vitamin D2 or ergocalciferol (which is predominantly obtained by dietary intake) [54]. Vitamin D2 and D3 are hydroxylated in the liver by vitamin D-25-hydroxylase to produce the major circulating form of vitamin D, 25-hydroxyvitamin D (25(OH)D) [55,56]. The serum concentration of 25(OH)D is one of the most reliable biomarkers of vitamin D status [56]. There are few foods with naturally occurring vitamin D and only 10–50% of the body’s vitamin D levels are obtained through dietary intake with the remainder being produced in the skin [57,58]. Vitamin D deficiency has been associated with the increased risk of cancer, obesity, osteoporosis, infectious and immune-mediated diseases, and cardiovascular disease [56]. It may also be involved with the onset of type 2 diabetes and impaired glucose metabolism. However, study results have been inconsistent. A large prospective case-control study found that dietary vitamin D intake was not significantly associated with the incidence of type 2 diabetes [59]. The Nurses’ Health Study found that no significant association between dietary vitamin D intake and type 2 diabetes [60]. However, a significant inverse relationship was observed between vitamin D supplementation and incident diabetes in women who consumed ≥400 UI/day of supplemental vitamin D [60]. These women had a 13% lower risk of developing diabetes, compared with those who consumed ≤100 UI/day of supplemental vitamin D [60]. A randomised controlled trial by Mitri et al. found that short-term cholecalciferol supplementation (2000 IU/day for 16 weeks), with or without calcium supplementation, improved β-cell function (as determined by a disposition index), insulin secretion, and attenuated the rise of HbA1c levels in adults at risk of type 2 diabetes [61]. In contrast, Gagnon et al. found that daily supplementation of 2000–6000 UI/day of cholecalciferol and calcium for six months resulted in no significant effect on insulin sensitivity (as determined by HOMA-S and Matsuda index), insulin secretion (as determined by insulinogenic index and C-peptide), and β-cell function (as determined by a disposition index), despite significant increase in circulating 25(OH)D [62]. A study of prediabetic individuals with vitamin D deficiency showed that 1200 IU/d of cholecalciferol and 500 mg of calcium for 16 weeks significantly increased mean serum 25(OH)D levels compared with the placebo. However, these did not improve insulin sensitivity (as determined by Stumvoll index and HOMA-IR), β-cell function (as determined by insulinogenic index), HbA1c, fasting glucose, or glucose tolerance [63]. Long-term vitamin D supplementation (2000 IU/week for five years) also had no significant effect on glucose metabolism or insulin resistance [64]. Therefore, evidence suggests that intake of vitamin D (either dietary or supplemental) has a limited effect on type 2 diabetes, glucose metabolism, and insulin resistance, irrespective of dosage or period of supplementation. Circulating vitamin D levels (25(OH)D) have been found to have an inverse association with the risk of type 2 diabetes in a meta-analysis of prospective studies [65]. Baseline serum 25(OH)D ≥50 nmol/L was significantly associated with decreased risk of type 2 diabetes [65]. Results from the study by Gao et al. also showed that circulating 25(OH)D had a positive association with insulin sensitivity (as determined by Matsuda index), a negative association with insulin resistance (as determined by HOMA-IR), and β-cell function (as determined by disposition Index) in women (but not men) with newly diagnosed type 2 diabetes [66].

The present study was the first to investigate the associations between habitual vitamin D intake and markers of glucose metabolism in individuals after an attack of AP. No significant associations were observed between dietary vitamin D intake and FPG, HOMA-IR, or HOMA-β. These results are consistent with evidence from other disease states, suggesting that vitamin D intake has little effect on diabetes risk and insulin traits; however, circulating serum vitamin D levels may indicate risk of NODAP. Sunlight exposure is the primary determinant of circulating 25(OH)D and overall vitamin D status; therefore, dietary intake may not reflect overall vitamin D status [57,58]. Vitamin D levels of AP patients have been found to be insufficient, deficient, or severely deficient in up to 40% of AP patients during hospital admission [67]. Serum 25(OH)D levels also decreased within the first two days of hospital admission [67]. Similarly, the prevalence of vitamin D insufficiency and deficiency was 28.5% and 56.2% in patients with AP, and these were associated with increased AP severity and risk of admission to the ICU [68]. Emerging evidence also demonstrates the perpetuation of low-grade inflammation long after the initial AP attack [65,69]. Therefore, the inflammatory state of AP may influence long-term fat-soluble vitamin status and may be associated with altered insulin traits. It is suggested that upregulation 1α-hydroxylase alters the synthesis of 1,25(OH)D by macrophages and tumour necrosis factor-α during inflammation, hence depleting the reservoir of 25(OH)D [67,70]. Based on the above arguments, further investigations on circulating vitamin D status and NODAP are warranted.

### 4.2. Water-Soluble Vitamins

Water-soluble vitamins are a group of structurally dissimilar, hydrophilic, organic compounds that include B vitamins and vitamin C [3,71]. Humans have evolutionarily lost the ability to endogenously synthesise most water-soluble vitamins (except for vitamin B3, which can be synthesised by gut bacteria in small quantities) [72,73]. Therefore, these vitamins must be obtained via dietary intake [71]. Water-soluble vitamins are not stored in large quantities throughout the body and are readily excreted through urine [3]. Therefore, short periods of inadequate water-soluble vitamin intake increases risk of vitamin deficiency [74]. Several potential causes of B vitamin deficiency include inadequate intake, increased requirements, malabsorption, drug [75].

Vitamin B3, also known as niacin, plays a role in energy metabolism, redox reactions, and reduce oxidative stress [76,77]. Dietary vitamin B3 is primarily in the form of nicotinic acid and nicotinamide; however some foods may contain small amounts of nicotinamide adenine dinucleotide (NAD) and nicotinamide adenine dinucleotide phosphate (NADP) [78]. Nicotinamide can also be derived from the amino acid tryptophan, therefore foods high in tryptophan are also considered sources of vitamin B3 [78]. Vitamin B3 has a broad spectrum of biological functions, including serving as a cofactor for redox reactions, a substrate for enzymes, and a ligand for purine receptors [78]. Vitamin B3 also inhibits the production of the pro-inflammatory cytokines (hence, reducing inflammation) [73,79]. Studies investigating associations between dietary intake of vitamin B3 and diabetes and insulin traits are scarce, with inconsistent results. Eshak et al. observed an inverse relationship between vitamin B3 intake and diabetes in men and women; however, this association became non-significant after adjusting for alcohol intake and magnesium intake [14]. A study by Mancini et al. observed that dietary patterns with a positive loading for vitamin B3 and magnesium reduced the risk of developing type 2 diabetes, concluding that intakes high in vitamin B3 and magnesium may have a protective effect against type 2 diabetes when consumed together [80]. Niacin therapy has also been investigated in individuals with type 2 diabetes. It was shown that ≤2.5 g/day of niacin, alone or combined with statins, was effective in reducing cardiovascular events in those with diabetes [81]. Glycaemic control in the study cohort was mildly impaired with increases in fasting glucose by up to 5% and a 0.3% increase in HbA1c. However, these results were transient and reversible with antidiabetic medications [81]. More recent studies have also suggested that niacin treatment has a more significant effect on glucose levels in individuals with diabetes and may increase the risk of developing diabetes. A meta-analysis of randomised controlled trials found a 34% increase in the risk of developing diabetes in individuals who received niacin therapy [82]. Consistent results were observed in the HPS2-THRIVE study, with a 55% increase in significant disturbances in glycaemic control for individuals with diabetes taking extended-release niacin (compared with the placebo group) [83]. Additionally, those in the treatment group also had a 32% proportional increase in the diagnosis of diabetes compared with the placebo group [83]. Overall, evidence suggests that dietary intake of vitamin B3 may have limited effects on type 2 diabetes, particularly in women. The use of niacin therapy and pharmacological doses of vitamin B3 appears to negatively impact glycaemic control.

The present study found that reduced dietary intake of vitamin B3 was significantly directly associated with HOMA-β. Specifically, with every 1% decrease in vitamin B3 intake, HOMA-β decreased by 1.35% in individuals with NODAP. Therefore, it appears that insulin secretion in individuals with NODAP may be improved by increased vitamin B3 intake. However, the mechanisms behind the observed results are unclear. Vitamin B3 (specifically nicotinic acid) is well known for regulating dyslipidaemia and its effects on cardiovascular disease [78,84,85,86]. These effects are mediated by agonistic action of nicotinic acid on nicotinic acid G-protein-coupled pathway receptor GPR109a [78]. In adipocytes, activation of GPR109a suppresses the release of free fatty acids from adipose tissue, reducing free fatty acid flux to the liver, hence reducing the synthesis of triglyceride and VLDL production by substrate depletion [79]. It is not yet clear whether the observed derangements in glycaemic control with high dose niacin therapy is a side effect of increased GRP109a activation; however, use of low dose niacin therapy and dietary intake of vitamin B3 appear to not impact glucose homeostasis [78,87,88]. Chronically elevated lipid and lipoprotein profiles are associated with glucose intolerance, insulin resistance, and the onset of type 2 diabetes by inhibiting insulin-mediated glucose transporters in skeletal muscle [89,90,91,92]. Therefore, there may be a link between the GPR109a receptor, improved lipid homeostasis, and improved insulin secretion (though further investigations are required to validate this hypothesis). A previous study by the COSMOS group observed associations between lipid metabolism and individuals with chronic hyperglycaemia after AP [93]. In that study, chronic hyperglycaemia was significantly associated with elevated serum triglyceride and glycerol levels (consistent with findings in individuals with type 2 diabetes), yet not free fatty acids or apolipoprotein-B levels. The study also found that insulin and HOMA-IR were associated with lipid metabolism in patients after AP [93]. These results highlight the abnormal lipid profile of patients with chronic hyperglycaemia after an attack of AP and suggest that there may be a potential role for triglyceride-lowering pharmacotherapy in reducing the risk of NODAP [93]. Dietary vitamin B3 may improve insulin secretion and abnormal lipid profile of individuals after AP, reducing the risk of NODAP. However, pharmacological doses of vitamin B3 may have detrimental effects on glycaemic control in individuals with [83] and without diabetes [82,83]. Therefore, well-designed clinical studies are warranted to investigate these associations in people after an attack of AP.

### 4.3. Limitations

There are several limitations to consider within the present study. First, a self-administered FFQ was used to ascertain habitual intake of vitamins. Therefore, the possibility of recall bias cannot be discounted due to requiring the respondent to recall their diet retrospectively. Additionally, intentional or accidental over- or under-reporting of portion sizes and/or food frequency may also impact the accuracy of data [94]. However, the EPIC-Norfolk FFQ has been extensively validated in various populations, providing a more accurate representation of long-term vitamin intake compared with other dietary assessment methods [33,95]. Second, due to limitations of the EPIC-Norfolk FFQ and FETA software, intake of vitamin K and other vitamers and supplement intake were not able to be assessed [33]. Future studies should consider investigating other vitamins and vitamers in individuals after AP. Third, it is possible that dietary changes were made after an attack of AP, altering participant nutrient intake. However, FFQ data were collected an average of 26 months after an AP attack and are representative of a participant’s habitual intake of the past 12 months. Further, participants were not provided nutrition advice or encouraged to change their diet. Forth, vitamins/vitamers were investigated in isolation. Therefore, interactions between vitamins and other dietary confounders were not assessed. It is well known that nutrients and other dietary factors influence the bioavailability and absorption of other vitamins and impact glycaemic control and insulin traits [96,97,98]. However, due to the relatively small sample size of the present study, accounting for each of these covariates would result in the overfitting of statistical models. Therefore, the use of average daily energy intake was used as a single covariate to account for most other dietary variables, along with other covariates (age, sex, V/S fat volume ratio, smoking status, alcohol intake, aetiology of AP, number of AP episodes, cholecystectomy, use of antidiabetic medications). The V/S fat volume ratio was also used instead of more commonly used markers of adiposity (BMI and waist circumference), as it is a more comprehensive measure of abdominal adiposity and metabolic risk [99,100]. It is also worth noting that the use of antidiabetic medications was exclusive to the T2DM group and could affect results. Therefore, antidiabetic medications were included in statistical models. This study also did not assess other possible confounders (e.g., inflammatory markers, intra-pancreatic fat deposition), which may affect glucose metabolism and insulin traits following AP [101,102,103,104,105,106]. Due to the relatively small sample size of the present study and results not being corrected for multiple testing, there is a risk of type I error. Therefore, associations uncovered in this study should be externally validated by studies with larger sample size and adjust data for multiplicity. Additionally, the study cohort was predominantly made up of men, and the percentage of men in the NODAP group was significantly higher than that of the NAP group. It has previously been found that men have higher risk of developing NODAP compared with women [107]. This may be attributed to genetic variation between men and women, or difference in lifestyle factors such as alcoholism or smoking [107]. Although the present study did not investigate differences in vitamin/vitamer intake by sex, we included sex as a covariate. Fifth, habitual vitamin intake was assessed in this study, which is not necessarily reflective of vitamin status, particularly in a population that may be prone to malabsorption (such as NODAP) [20]. The vitamin status of an individual may affect insulin traits and glycaemic control; therefore, future studies should investigate vitamin levels in a post-pancreatitis population using more advanced assessment methods. Sixth, in this study, HOMA indices were used as markers of glucose metabolism. HOMA indices have been found to be a reliable indicator of glucose metabolism in individuals with impaired glucose tolerance, type 2 diabetes, and normoglycaemia [108]. However, HOMA indices may have limited accuracy in smaller study samples [108,109]. Therefore, future studies may consider using the hyperinsulinaemic-euglycaemic glucose clamp (the ‘gold standard’ for assessment of insulin traits) in exploring the associations between vitamin intake and glucose metabolism after an attack of AP [110]. Last, as this study had a cross-sectional design, a causal relationship between vitamin intake and NODAP cannot be established. However, in the first study investigating associations between vitamin intake and NODAP, we have provided insights that may assist the design of future studies of habitual vitamin intake in individuals after an attack of AP.

## 5. Conclusions

Of the 14 water-soluble and fat-soluble vitamins and vitamers investigated in the present study, habitual intake of α-carotene, β-carotene, total carotenoids and vitamin B3 was significantly directly associated with HOMA-β. The findings provide first evidence that intake of these vitamins/vitamers may have a role in NODAP. Longitudinal cohort and randomised controlled trials are now warranted to investigate causal relationships between these vitamins and NODAP as well as to unveil the mechanisms behind these associations, providing further evidence for nutrition interventions for individuals after an attack of AP.

## Figures and Tables

**Table 1:** Characteristics of the study cohort.

Characteristic	Total	NODAP	T2DM	NAP	*p* *
	(*n* = 106)	(*n* = 37)	(*n* = 37)	(*n* = 32)	
Age (years)	56.1 (14.5)	58.9 (14.4)	57.2 (15.0)	51.6 (13.3)	0.094
Men, *n* (%)	69 (65.1)	26 (70.3)	28 (75.7)	15 (46.9)	**0.031**
Daily energy intake (kcal)	1686 (609)	1776 (692)	1728 (534)	1534 (575)	0.226
V/S fat volume ratio	0.77 (0.43)	0.81(0.40)	0.87 (0.46)	0.61 (0.40)	**0.035**
Alcohol intake (g/day)	11.08 (17.91)	13.43 (21.90)	8.65 (13.05)	11.08 (17.70)	0.527
Tobacco smoking					0.052
Yes	23 (21.7)	10 (27.0)	4 (10.8)	9 (28.2)	
No	82 (77.3)	27 (72.9)	32 (86.5)	23 (71.9)	
Aetiology of AP					0.563
Biliary	40 (37.7)	14 (37.8)	14 (37.8)	12 (37.5)	
Non-biliary	66 (62.3)	23 (62.1)	23 (62.1)	20 (62.5)	
Number of AP episodes	1.85 (2.77)	2.27 (3.76)	1.43 (1.04)	1.84 (2.82)	0.434
Cholecystectomy					0.538
Yes	39 (36.8)	13 (35.1)	12 (32.4)	14 (43.8)	
No	66 (62.3)	24 (64.9)	25 (67.6)	17 (53.1)	
Anti-diabetic medication use					<**0.001**
None	92 (86.8)	37 (100)	23 (62.2)	32 (100)	
Oral medication	8 (7.5)	0 (0)	8 (21.6)	0 (0)	
Insulin	6 (5.7)	0 (0)	6 (16.2)	0 (0)	
Fasting plasma glucose (mmol/L)	5.86 (1.74)	5.86 (0.92)	6.61 (2.55)	4.96 (0.34)	<**0.001**
HOMA-β (%)	106.97 (56.87)	95.74 (45.63)	103.24 (57.12)	125.07 (65.87)	0.098
HOMA-IR (mIU/L-mmol/L)	1.76 (1.28)	1.73 (1.29)	1.97 (1.31)	1.55 (1.22)	0.408
Fasting insulin (mU/L)	16.68 (36.01)	12.98 (9.96)	24.62 (59.95)	12.15 (10.27)	0.277

Abbreviations: AP = Acute pancreatitis. HOMA-IR = homeostasis model assessment of insulin resistance. HOMA-β = homeostasis model assessment of β-cell dysfunction. NAP = Normoglycaemia after acute pancreatitis. NODAP = New-onset diabetes or prediabetes after acute pancreatitis. T2DM = Type 2 diabetes or prediabetes prior to acute pancreatitis. V/S fat volume ratio = Visceral to subcutaneous fat volume ratio. Data are presented as mean (standard deviation) or frequency(percentage). * *p* values were calculated from one way ANOVA and represent the difference between groups. Significance was set at *p* < 0.05. Significant values are shown in bold.

**Table 2:** Associations between habitual fat-soluble vitamin/vitamer intake and fasting plasma glucose in the study groups.

Vitamin	Model	NAP					T2DM					NODAP				
		R²	Unstandardised	*p*	95% CI		R²	Unstandardised	*p*	95% CI		R²	Unstandardised	*p*	95% CI	
			B		Lower	Upper		B		Lower	Upper		B		Lower	Upper
α-Carotene (µg)	1	0.025	−0.116	0.400	−0.393	0.162	0.062	−0.839	0.149	−1.993	0.315	0.032	−0.341	0.292	−0.987	0.306
	2	0.157	−0.083	0.571	−0.381	0.215	0.085	−0.887	0.206	−2.287	0.514	0.268	−0.486	0.110	−1.089	0.116
	3	0.233	−0.107	0.458	−0.400	0.186	0.105	−0.729	0.317	−2.194	0.736	0.269	−0.507	0.115	−1.145	0.131
	4	0.233	−0.111	0.510	−0.456	0.233	0.260	−0.334	0.638	−1.776	1.107	0.298	−0.478	0.146	−1.134	0.177
	5	0.247	−0.137	0.488	−0.543	0.269	0.533	0.266	0.690	−1.098	1.630	0.330	−0.474	0.167	−1.159	0.211
β-Carotene (µg)	1	0.039	−0.169	0.293	−0.491	0.154	0.004	−0.339	0.716	−2.215	1.537	0.037	−0.633	0.252	−1.737	0.471
	2	0.162	−0.117	0.499	−0.467	0.234	0.034	−0.107	0.926	−2.446	2.232	0.254	−0.715	0.164	−1.737	0.306
	3	0.229	−0.113	0.505	−0.456	0.231	0.075	0.283	0.814	−2.152	2.719	0.254	−0.714	0.176	−1.765	0.337
	4	0.231	−0.124	0.538	−0.536	0.288	0.264	0.735	0.528	−1.622	3.092	0.286	−0.697	0.201	−1.789	0.394
	5	0.243	−0.154	0.539	−0.668	0.361	0.556	1.230	0.248	−0.918	3.378	0.324	−0.751	0.193	−1.905	0.403
Retinol (µg)	1	0.089	0.296	0.110	−0.071	0.663	0.024	−0.863	0.370	−2.794	1.068	0.083	0.666	0.084	−0.094	1.425
	2	0.221	0.318	0.134	−0.104	0.740	0.056	−0.943	0.413	−3.261	1.375	0.250	0.582	0.180	−0.282	1.446
	3	0.321	0.385	0.064	−0.025	0.794	0.081	−0.604	0.618	−3.056	1.847	0.257	0.633	0.160	−0.263	1.530
	4	0.350	0.458	**0.046**	0.009	0.907	0.260	−0.540	0.635	−2.851	1.771	0.289	0.652	0.184	−0.328	1.632
	5	0.387	0.568	**0.038**	0.033	1.103	0.538	−0.672	0.520	−2.803	1.458	0.307	0.545	0.309	−0.535	1.624
Total carotene (µg)	1	0.037	−0.168	0.308	−0.499	0.163	0.011	−0.585	0.549	−2.554	1.383	0.043	−0.690	0.218	−1.806	0.426
	2	0.161	−0.117	0.516	−0.481	0.248	0.037	−0.376	0.750	−2.767	2.014	0.262	−0.790	0.128	−1.821	0.241
	3	0.229	−0.117	0.506	−0.474	0.241	0.073	0.045	0.971	−2.477	2.568	0.262	−0.794	0.138	−1.859	0.271
	4	0.231	−0.127	0.542	−0.551	0.298	0.256	0.377	0.748	−2.006	2.760	0.294	−0.771	0.161	−1.866	0.325
	5	0.243	−0.160	0.536	−0.692	0.372	0.537	0.657	0.533	−1.490	2.804	0.333	−0.831	0.153	−1.991	0.330
Total retinol equivalents (µg)	1	0.013	−0.115	0.541	−0.496	0.266	0.058	−1.812	0.162	−4.386	0.762	0.014	0.471	0.492	−0.904	1.845
	2	0.149	−0.062	0.769	−0.492	0.368	0.082	−1.960	0.219	−5.150	1.230	0.207	0.133	0.858	−1.374	1.640
	3	0.216	−0.038	0.853	−0.462	0.385	0.098	−1.515	0.377	−4.973	1.942	0.209	0.191	0.808	−1.399	1.781
	4	0.218	−0.021	0.930	−0.498	0.457	0.272	−1.309	0.414	−4.550	1.931	0.247	0.300	0.735	−1.493	2.093
	5	0.227	−0.018	0.955	−0.689	0.652	0.541	−1.085	0.446	−3.980	1.809	0.278	0.125	0.898	−1.855	2.104
Vitamin D (µg)	1	0.101	0.394	0.087	−0.061	0.849	0.016	−1.374	0.468	−5.184	2.436	0.009	0.276	0.587	−0.746	1.299
	2	0.182	0.314	0.304	−0.302	0.931	0.040	−1.025	0.651	−5.610	3.559	0.213	−0.341	0.603	−1.663	0.981
	3	0.262	0.365	0.223	−0.237	0.967	0.078	−0.858	0.705	−5.447	3.731	0.214	−0.343	0.606	−1.687	1.001
	4	0.266	0.373	0.238	−0.265	1.011	0.254	−0.225	0.917	−4.595	4.145	0.256	−0.454	0.504	−1.828	0.919
	5	0.276	0.375	0.271	−0.318	1.069	0.533	0.908	0.659	−3.295	5.110	0.304	−0.704	0.332	−2.167	0.760
Vitamin E (mg)	1	0.079	−0.429	0.133	−0.998	0.139	0.025	−1.469	0.368	−4.742	1.804	0.053	0.888	0.169	−0.396	2.173
	2	0.280	−0.691	**0.040**	−1.349	−0.033	0.043	−1.594	0.599	−7.725	4.537	0.226	−1.049	0.373	−3.415	1.317
	3	0.308	−0.600	0.085	−1.288	0.088	0.080	−1.369	0.652	−7.506	4.768	0.226	−1.052	0.395	−3.540	1.436
	4	0.308	−0.597	0.104	−1.325	0.132	0.277	−2.726	0.357	−8.697	3.245	0.277	−1.454	0.254	−4.011	1.103
	5	0.320	−0.673	0.124	−1.549	0.202	0.529	−0.156	0.959	−6.341	6.029	0.299	−1.183	0.387	−3.947	1.582

Abbreviations: NAP = Normoglycaemia after acute pancreatitis. NODAP = New-onset diabetes or prediabetes after acute pancreatitis. T2DM = Type 2 diabetes or prediabetes prior to acute pancreatitis. 95% CI = 95% confidence interval. Data are presented as R^2^ values (from crude analysis), unstandardised B, *p* values (from linear regression) and 95% confidence intervals. All the variables were log-transformed. Model 1: unadjusted model. Model 2: age, sex, daily energy intake. Model 3: age, sex, daily energy intake, V/S fat volume ratio. Model 4: age, sex, daily energy intake, V/S fat volume ratio, alcohol intake, smoking status. Model 5: age, sex, daily energy intake, V/S fat volume ratio, alcohol intake, smoking status, aetiology of AP, number of AP episodes, cholecystectomy, use of antidiabetic medications. Significance was set at *p* < 0.05. Significant values are shown in bold.

**Table 3:** Associations between habitual fat-soluble vitamin/vitamer intake and HOMA-β in the study groups.

Vitamin	Model	NAP					T2DM					NODAP				
		R²	Unstandardised	*p*	95% CI		R²	Unstandardised	*p*	95% CI		R²	Unstandardised	*p*	95% CI	
			B		Lower	Upper		B		Lower	Upper		B		Lower	Upper
α-Carotene (µg)	1	0.085	42.654	0.118	−11.525	96.833	0.026	12.369	0.380	−15.993	40.730	0.122	33.125	** 0.034 **	2.563	63.688
	2	0.097	46.408	0.137	−15.817	108.633	0.067	11.596	0.509	−23.970	47.162	0.144	31.487	0.056	−0.863	63.836
	3	0.120	49.019	0.123	−14.212	112.250	0.071	9.963	0.592	−27.775	47.701	0.161	35.211	** 0.042 **	1.280	69.143
	4	0.149	42.815	0.238	−30.423	116.053	0.167	2.010	0.916	−37.064	41.083	0.265	39.096	** 0.023 **	5.803	72.390
	5	0.253	46.748	0.245	−34.802	128.298	0.228	−2.449	0.918	−51.174	46.277	0.400	41.584	** 0.013 **	9.383	73.785
β-Carotene (µg)	1	0.046	36.574	0.258	−28.244	101.392	0.017	16.025	0.479	−29.657	61.706	0.075	44.684	0.100	−9.036	98.405
	2	0.062	41.825	0.260	−32.875	116.526	0.059	12.900	0.648	−44.502	70.302	0.111	43.746	0.117	−11.620	99.112
	3	0.075	41.472	0.271	−34.421	117.365	0.064	9.766	0.744	−51.147	70.678	0.117	45.524	0.112	−11.213	102.261
	4	0.105	23.914	0.586	−65.736	113.565	0.166	−0.629	0.984	−65.396	64.139	0.235	57.657	** 0.044 **	1.591	113.723
	5	0.234	48.647	0.341	−55.638	152.933	0.229	−6.627	0.859	−83.196	69.942	0.360	60.440	** 0.035 **	4.672	116.209
Retinol (µg)	1	0.028	33.740	0.374	−42.742	110.222	0.001	4.373	0.856	−44.366	53.112	0.000	−2.031	0.917	−41.393	37.331
	2	0.048	44.359	0.341	−49.691	138.410	0.060	−14.040	0.628	−72.872	44.791	0.051	−14.781	0.537	−63.044	33.482
	3	0.055	40.772	0.396	−56.658	138.202	0.076	−20.059	0.515	−82.446	42.329	0.051	−14.404	0.564	−64.723	35.916
	4	0.124	45.228	0.382	−59.861	150.317	0.183	−20.912	0.488	−82.218	40.394	0.119	−0.939	0.972	−55.105	53.228
	5	0.235	56.332	0.340	−64.138	176.803	0.246	−22.664	0.498	−91.238	45.911	0.249	−16.912	0.539	−72.685	38.860
Total carotene (µg)	1	0.053	40.540	0.220	−25.715	106.796	0.013	15.205	0.527	−33.323	63.734	0.090	49.461	0.072	−4.580	103.502
	2	0.072	47.608	0.217	−29.735	124.951	0.058	12.827	0.667	−47.733	73.387	0.124	48.182	0.088	−7.591	103.956
	3	0.086	47.633	0.223	−30.889	126.155	0.063	9.133	0.775	−55.814	74.081	0.132	50.738	0.081	−6.625	108.102
	4	0.112	31.256	0.488	−60.620	123.132	0.166	2.405	0.942	−65.244	70.054	0.241	59.620	** 0.039 **	3.231	116.009
	5	0.247	58.189	0.269	−48.786	165.163	0.228	−0.725	0.985	−79.302	77.852	0.368	63.178	** 0.029 **	7.069	119.286
Total retinol equivalents (µg)	1	0.043	41.583	0.269	−33.984	117.150	0.027	29.823	0.371	−37.247	96.893	0.007	16.417	0.630	−52.062	84.896
	2	0.061	50.225	0.267	−40.850	141.300	0.055	13.194	0.755	−72.675	99.062	0.040	5.249	0.897	−77.085	87.583
	3	0.070	48.395	0.293	−44.571	141.360	0.061	6.655	0.885	−86.884	100.194	0.041	8.065	0.851	−78.824	94.955
	4	0.111	34.100	0.498	−68.523	136.723	0.166	1.319	0.977	−91.619	94.257	0.149	47.023	0.318	−47.582	141.628
	5	0.265	84.330	0.196	−47.518	216.178	0.228	−2.039	0.967	−102.177	98.099	0.254	36.814	0.456	−63.106	136.734
Vitamin D (µg)	1	0.003	13.246	0.781	−83.424	109.916	0.025	−44.015	0.390	−146.965	58.935	0.056	35.164	0.159	−14.385	84.712
	2	0.024	36.265	0.587	−99.552	172.082	0.116	−80.625	0.170	−197.999	36.749	0.127	60.927	0.082	−8.187	130.041
	3	0.035	32.472	0.634	−106.445	171.388	0.128	−82.399	0.167	−201.624	36.826	0.128	60.837	0.087	−9.439	131.113
	4	0.109	43.550	0.531	−98.202	185.303	0.291	−115.998	0.051	−232.456	0.460	0.192	56.521	0.114	−14.500	127.542
	5	0.216	48.852	0.491	−96.631	194.335	0.347	−122.811	0.071	−256.989	11.367	0.275	42.089	0.254	−32.052	116.231
Vitamin E (mg)	1	0.003	16.183	0.783	−103.121	135.488	0.066	61.903	0.156	−24.999	148.806	0.014	22.958	0.478	−42.091	88.007
	2	0.016	23.462	0.758	−131.794	178.719	0.107	100.700	0.205	−58.552	259.953	0.048	34.906	0.589	−95.343	165.154
	3	0.027	11.989	0.882	−152.544	176.522	0.113	98.254	0.225	−64.290	260.798	0.052	40.872	0.546	−95.836	177.580
	4	0.092	2.743	0.973	−165.515	171.002	0.225	105.902	0.191	−56.399	268.203	0.132	44.707	0.516	−94.467	183.881
	5	0.210	52.021	0.574	−138.296	242.338	0.258	92.114	0.374	−119.097	303.326	0.279	83.141	0.230	−56.011	222.293

Abbreviations: HOMA-β = homeostasis model assessment β-cell function. NAP = Normoglycaemia after acute pancreatitis. NODAP = New-onset diabetes or prediabetes after acute pancreatitis. T2DM = Type 2 diabetes or prediabetes prior to acute pancreatitis. 95% CI = 95% confidence interval. Data are presented as R^2^ values (from crude analysis), unstandardised B, *p* values (from linear regression) and 95% confidence intervals. All the variables were log-transformed. Model 1: unadjusted model. Model 2: age, sex, daily energy intake. Model 3: age, sex, daily energy intake, V/S fat volume ratio. Model 4: age, sex, daily energy intake, V/S fat volume ratio, alcohol intake, smoking status. Model 5: age, sex, daily energy intake, V/S fat volume ratio, alcohol intake, smoking status, aetiology of AP, number of AP episodes, cholecystectomy, use of antidiabetic medications. Significance was set at *p* < 0.05. Significant values are shown in bold.

**Table 4:** Associations between habitual water-soluble vitamin intake and fasting plasma glucose in the study groups.

Vitamin	Model	NAP					T2DM					NODAP				
		R²	Unstandardised	*p*	95% CI		R²	Unstandardised	*p*	95% CI		R²	Unstandardised	*p*	95% CI	
			B		Lower	Upper		B		Lower	Upper		B		Lower	Upper
Vitamin B1 (mg)	1	0.002	−0.084	0.828	−0.867	0.700	0.089	−3.682	0.081	−7.847	0.483	0.052	1.227	0.177	−0.579	3.033
	2	0.181	−0.573	0.313	−1.716	0.571	0.121	−5.077	0.096	−11.104	0.950	0.216	−0.856	0.537	−3.649	1.937
	3	0.221	−0.282	0.648	−1.543	0.978	0.143	−4.615	0.136	−10.774	1.545	0.217	−0.875	0.535	−3.718	1.967
	4	0.222	−0.252	0.712	−1.648	1.144	0.368	−6.024	**0.036**	−11.612	−0.437	0.265	−1.321	0.365	−4.257	1.615
	5	0.234	−0.337	0.677	−2.003	1.329	0.573	−3.930	0.137	−9.206	1.346	0.311	−1.711	0.271	−4.836	1.414
Vitamin B2 (mg)	1	0.005	0.144	0.716	−0.656	0.943	0.106	−4.251	0.056	−8.613	0.111	0.091	1.965	0.069	−0.161	4.091
	2	0.146	0.019	0.972	−1.058	1.095	0.176	−6.653	**0.030**	−12.614	−0.691	0.211	0.718	0.648	−2.451	3.888
	3	0.219	0.193	0.716	−0.888	1.273	0.200	−6.313	**0.041**	−12.347	−0.279	0.213	0.769	0.632	−2.474	4.011
	4	0.221	0.184	0.741	−0.952	1.319	0.357	−5.771	**0.046**	−11.439	−0.104	0.275	2.198	0.272	−1.820	6.216
	5	0.229	0.194	0.744	−1.030	1.418	0.593	−5.056	0.070	−10.560	0.448	0.301	2.103	0.368	−2.610	6.816
Vitamin B3 (mg)	1	0.042	−0.442	0.277	−1.259	0.375	0.055	−3.346	0.173	−8.237	1.544	0.029	0.808	0.316	−0.804	2.421
	2	0.295	−1.100	**0.030**	−2.086	−0.115	0.095	−4.751	0.167	−11.594	2.092	0.224	−0.943	0.400	−3.194	1.308
	3	0.323	−0.972	0.062	−1.995	0.051	0.132	−4.694	0.170	−11.517	2.130	0.224	−0.930	0.415	−3.225	1.366
	4	0.331	−1.014	0.066	−2.098	0.071	0.276	−3.074	0.362	−9.881	3.733	0.255	−0.853	0.509	−3.459	1.754
	5	0.334	−1.058	0.096	−2.324	0.208	0.531	−1.020	0.750	−7.572	5.532	0.291	−0.931	0.488	−3.650	1.789
Vitamin B6 (mg)	1	0.019	−0.320	0.466	−1.208	0.567	0.051	−3.222	0.192	−8.141	1.696	0.026	0.916	0.336	−0.992	2.824
	2	0.242	−1.114	0.088	−2.404	0.176	0.098	−5.976	0.154	−14.314	2.362	0.239	−1.504	0.246	−4.096	1.089
	3	0.274	−0.920	0.172	−2.267	0.428	0.132	−5.717	0.172	−14.064	2.631	0.239	−1.492	0.261	−4.150	1.167
	4	0.275	−0.910	0.200	−2.337	0.517	0.280	−3.967	0.322	−12.036	4.101	0.268	−1.396	0.333	−4.297	1.505
	5	0.291	−1.025	0.205	−2.661	0.611	0.565	−4.942	0.183	−12.394	2.510	0.301	−1.355	0.365	−4.380	1.669
Vitamin B9 (µg)	1	0.016	−0.192	0.502	−0.770	0.386	0.002	−0.430	0.813	−4.103	3.242	0.003	0.270	0.742	−1.381	1.921
	2	0.174	−0.314	0.365	−1.015	0.387	0.035	0.530	0.836	−4.650	5.711	0.258	−1.391	0.143	−3.277	0.495
	3	0.237	−0.280	0.411	−0.971	0.411	0.076	0.727	0.776	−4.450	5.903	0.259	−1.385	0.151	−3.304	0.535
	4	0.237	−0.276	0.454	−1.028	0.475	0.254	0.251	0.917	−4.609	5.110	0.274	−1.182	0.284	−3.396	1.032
	5	0.259	−0.413	0.374	−1.362	0.536	0.530	−0.327	0.879	−4.715	4.061	0.316	−1.381	0.238	−3.728	0.967
Vitamin B12 (µg)	1	0.041	0.281	0.283	−0.245	0.808	0.270	−5.259	**0.001**	−8.326	−2.193	0.055	0.926	0.164	−0.397	2.250
	2	0.153	0.140	0.655	−0.497	0.777	0.310	−5.895	**0.002**	−9.372	−2.418	0.212	0.387	0.634	−1.253	2.026
	3	0.233	0.231	0.457	−0.400	0.863	0.319	−5.676	**0.003**	−9.262	−2.090	0.214	0.420	0.615	−1.263	2.102
	4	0.233	0.223	0.502	−0.455	0.902	0.384	−4.698	**0.024**	−8.723	−0.674	0.250	0.430	0.630	−1.378	2.239
	5	0.240	0.213	0.573	−0.564	0.990	0.593	−3.437	0.069	−7.169	0.295	0.281	0.318	0.734	−1.589	2.225
Vitamin C (mg)	1	0.052	−0.236	0.223	−0.625	0.152	0.005	−0.510	0.677	−2.974	1.955	0.005	−0.258	0.673	−1.486	0.971
	2	0.201	−0.284	0.202	−0.730	0.162	0.034	−0.129	0.926	−2.935	2.677	0.242	−0.708	0.229	−1.883	0.468
	3	0.255	−0.245	0.267	−0.690	0.200	0.074	0.197	0.889	−2.665	3.058	0.242	−0.703	0.240	−1.900	0.493
	4	0.258	−0.268	0.284	−0.773	0.238	0.253	−0.088	0.947	−2.778	2.602	0.284	−0.756	0.214	−1.973	0.461
	5	0.296	−0.441	0.190	−1.119	0.238	0.540	−0.866	0.466	−3.282	1.551	0.341	−1.034	0.127	−2.382	0.315

Abbreviations: NAP = Normoglycaemia after acute pancreatitis. NODAP = New-onset diabetes or prediabetes after acute pancreatitis. T2DM = Type 2 diabetes or prediabetes prior to acute pancreatitis. 95% CI = 95% confidence interval. Data are presented as R^2^ values (from crude analysis), unstandardised B, *p* values (from linear regression) and 95% confidence intervals. All the variables were log-transformed. Model 1: unadjusted model. Model 2: age, sex, daily energy intake. Model 3: age, sex, daily energy intake, V/S fat volume ratio. Model 4: age, sex, daily energy intake, V/S fat volume ratio, alcohol intake, smoking status. Model 5: age, sex, daily energy intake, V/S fat volume ratio, alcohol intake, smoking status, aetiology of AP, number of AP episodes, cholecystectomy, use of antidiabetic medications. Significance was set at *p* < 0.05. Significant values are shown in bold.

**Table 5:** Associations between habitual water-soluble vitamin intake and HOMA-β in the study groups.

Vitamin	Model	NAP					T2DM					NODAP				
		R²	Unstandardised	*p*	95% CI		R²	Unstandardised	*p*	95% CI		R²	Unstandardised	*p*	95% CI	
			B		Lower	Upper		B		Lower	Upper		B		Lower	Upper
Vitamin B1 (mg)	1	0.011	42.743	0.582	−114.512	199.998	0.010	32.893	0.577	−86.282	152.067	0.055	62.654	0.164	−26.869	152.178
	2	0.051	121.755	0.322	−126.508	370.018	0.053	19.778	0.811	−148.017	187.573	0.131	131.536	0.076	−14.439	277.511
	3	0.052	111.205	0.421	−169.103	391.512	0.061	13.557	0.873	−159.371	186.485	0.131	131.084	0.082	−17.611	279.778
	4	0.109	94.553	0.522	−206.688	395.793	0.170	28.241	0.739	−144.743	201.226	0.226	146.635	0.054	−2.996	296.265
	5	0.223	131.169	0.427	−207.308	469.646	0.234	40.247	0.677	−158.402	238.895	0.314	127.934	0.102	−26.982	282.850
Vitamin B2 (mg)	1	0.023	62.837	0.427	−96.935	222.609	0.059	76.867	0.180	−37.390	191.124	0.000	−1.518	0.978	−112.231	109.196
	2	0.068	135.116	0.231	−91.641	361.873	0.094	88.374	0.271	−72.907	249.655	0.042	−25.655	0.765	−199.099	147.790
	3	0.073	126.599	0.282	−110.736	363.935	0.099	85.231	0.298	−79.789	250.251	0.043	−23.939	0.785	−201.522	153.644
	4	0.140	128.952	0.278	−111.612	369.517	0.192	69.896	0.390	−94.709	234.501	0.131	69.627	0.519	−148.701	287.955
	5	0.233	119.315	0.347	−139.643	378.273	0.263	95.159	0.340	−107.822	298.141	0.238	10.611	0.930	−233.651	254.873
Vitamin B3 (mg)	1	0.025	68.587	0.405	−97.535	234.708	0.013	41.105	0.536	−92.863	175.072	0.032	42.527	0.287	−37.389	122.443
	2	0.076	144.887	0.202	−82.670	372.444	0.057	35.967	0.700	−153.789	225.723	0.079	70.447	0.247	−51.283	192.178
	3	0.079	136.725	0.253	−103.996	377.447	0.065	34.328	0.718	−158.689	227.345	0.082	71.752	0.247	−52.247	195.752
	4	0.169	167.720	0.168	−76.085	411.526	0.170	−32.271	0.750	−238.848	174.306	0.245	140.586	**0.035**	10.330	270.842
	5	0.342	249.197	0.054	−4.603	502.997	0.232	−38.662	0.737	−275.291	197.966	0.352	134.901	**0.041**	5.816	263.985
Vitamin B6 (mg)	1	0.029	79.794	0.367	−98.301	257.889	0.025	53.354	0.384	−70.034	176.742	0.004	18.678	0.695	−77.091	114.447
	2	0.106	222.157	0.118	−60.324	504.637	0.068	70.571	0.488	−135.447	276.589	0.040	14.355	0.841	−130.214	158.924
	3	0.107	215.652	0.153	−85.765	517.070	0.076	68.214	0.510	−141.538	277.965	0.042	16.470	0.822	−131.676	164.616
	4	0.164	215.652	0.153	−85.765	517.070	0.170	34.555	0.741	−178.907	248.017	0.138	61.693	0.426	−94.618	218.004
	5	0.283	240.804	0.145	−91.028	572.636	0.240	70.489	0.572	−185.510	326.487	0.259	65.964	0.388	−88.546	220.475
Vitamin B9 (µg)	1	0.030	52.984	0.356	−62.696	168.665	0.017	32.051	0.479	−59.229	123.332	0.016	30.339	0.454	−51.071	111.750
	2	0.066	87.722	0.240	−62.516	237.960	0.054	16.610	0.799	−115.882	149.103	0.057	40.811	0.437	−64.747	146.369
	3	0.076	84.973	0.264	−68.271	238.217	0.062	15.355	0.817	−119.424	150.133	0.059	41.402	0.438	−65.998	148.802
	4	0.120	65.458	0.413	−97.326	228.241	0.171	23.043	0.724	−110.081	156.167	0.173	79.214	0.178	−38.046	196.473
	5	0.235	91.910	0.335	−102.621	286.441	0.230	19.129	0.801	−137.147	175.405	0.284	75.258	0.206	−44.048	194.564
Vitamin B12 (µg)	1	0.000	−3.524	0.947	−111.910	104.863	0.070	66.147	0.144	−23.970	156.263	0.002	8.979	0.789	−58.525	76.483
	2	0.012	5.736	0.933	−133.002	144.475	0.094	56.737	0.272	−47.021	160.494	0.039	0.995	0.982	−88.885	90.874
	3	0.026	5.736	0.933	−133.002	144.475	0.097	53.918	0.312	−53.464	161.300	0.040	2.305	0.960	−89.975	94.585
	4	0.094	−1.786	0.980	−145.308	141.736	0.169	17.344	0.780	−109.359	144.048	0.130	29.526	0.537	−67.152	126.205
	5	0.197	9.464	0.903	−151.686	170.615	0.229	13.246	0.844	−125.424	151.916	0.241	17.287	0.718	−79.974	114.548
Vitamin C (mg)	1	0.049	45.996	0.240	−32.526	124.519	0.020	24.530	0.440	−39.475	88.536	0.068	46.339	0.120	−12.699	105.378
	2	0.078	62.628	0.194	−34.000	159.256	0.064	22.657	0.544	−53.029	98.344	0.120	52.901	0.096	−9.984	115.786
	3	0.085	59.848	0.226	−39.561	159.257	0.070	19.904	0.606	−58.556	98.364	0.122	53.264	0.099	−10.682	117.209
	4	0.116	41.520	0.447	−69.754	152.795	0.186	31.218	0.453	−53.300	115.736	0.183	47.746	0.141	−16.774	112.266
	5	0.236	67.759	0.332	−74.766	210.283	0.259	40.954	0.367	−51.682	133.591	0.290	46.795	0.178	−22.697	116.288

Abbreviations: HOMA-β = homeostasis model assessment β-cell function. NAP = Normoglycaemia after acute pancreatitis. NODAP = New-onset diabetes or prediabetes after acute pancreatitis. T2DM = Type 2 diabetes or prediabetes prior to acute pancreatitis. 95% CI = 95% confidence interval. Data are presented as R^2^ values (from crude analysis), unstandardised B, *p* values (from linear regression) and 95% confidence intervals. All the variables were log-transformed. Model 1: unadjusted model. Model 2: age, sex, daily energy intake. Model 3: age, sex, daily energy intake, V/S fat volume ratio. Model 4: age, sex, daily energy intake, V/S fat volume ratio, alcohol intake, smoking status. Model 5: age, sex, daily energy intake, V/S fat volume ratio, alcohol intake, smoking status, aetiology of AP, number of AP episodes, cholecystectomy, use of antidiabetic medications. Significance was set at *p* < 0.05. Significant values are shown in bold.

## Data Availability

The data are not publicly available due to the ethical conduct in human research regulations.

## Supplementary Materials

The following supporting information can be downloaded at: https://www.mdpi.com/article/10.3390/nu14071480/s1, Table S1: Associations between habitual fat-soluble vitamin/vitamer intake and HOMA-IR in the study groups, Table S2: Associations between habitual water-soluble vitamin intake and HOMA-IR in the study groups.
