# Supplementary material for: Relationship between Habitual Intake of Vitamins and New-Onset Prediabetes/Diabetes after Acute Pancreatitis
Source: Nutrients. 2022 Apr 1;14(7):1480. doi: 10.3390/nu14071480 (PMC9003206; doi:10.3390/nu14071480)
Supplement: Supplementary file 1 [file nutrients-14-01480-s001.zip › nutrients-1621860-supplementary.pdf]

Supplementary materials

Supplementary Table S1. Associations between habitual fat-soluble vitamin/vitamer intake and HOMA-IR in the study groups

Vitamin	Model	NAP					T2DM					NODAP				
		R^2	Unstandardised B	*p*	95% CI Lower	95% CI Upper	R^2	Unstandardised B	*p*	95% CI Lower	95% CI Upper	R^2	Unstandardised B	*p*	95% CI Lower	95% CI Upper
α-Carotene (μg)	1	0.058	0.654	0.200	-0.366	1.673	0.005	-0.131	0.694	-0.804	0.543	0.039	0.533	0.240	-0.372	1.437
	2	0.068	0.735	0.209	-0.437	1.907	0.139	-0.441	0.269	-1.244	0.361	0.175	0.368	0.411	-0.531	1.267
	3	0.077	0.767	0.200	-0.433	1.967	0.145	-0.395	0.349	-1.246	0.456	0.200	0.496	0.289	-0.442	1.434
	4	0.111	0.659	0.336	-0.729	2.046	0.202	-0.496	0.266	-1.395	0.402	0.293	0.620	0.180	-0.304	1.544
	5	0.232	0.709	0.345	-0.823	2.241	0.405	-0.187	0.701	-1.192	0.818	0.372	0.660	0.158	-0.273	1.592
β-Carotene (μg)	1	0.023	0.481	0.424	-0.735	1.697	0.002	0.141	0.792	-0.940	1.222	0.021	0.669	0.391	-0.896	2.234
	2	0.034	0.587	0.398	-0.818	1.993	0.101	-0.181	0.780	-1.500	1.137	0.172	0.569	0.449	-0.943	2.081
	3	0.039	0.583	0.409	-0.851	2.018	0.115	-0.061	0.929	-1.453	1.331	0.190	0.660	0.388	-0.878	2.197
	4	0.076	0.234	0.777	-1.455	1.922	0.166	-0.336	0.653	-1.858	1.187	0.290	0.985	0.198	-0.545	2.514
	5	0.215	0.659	0.490	-1.299	2.617	0.402	-0.151	0.845	-1.735	1.434	0.363	1.009	0.199	-0.565	2.583
Retinol (μg)	1	0.053	0.856	0.221	-0.544	2.256	0.007	-0.258	0.648	-1.400	0.884	0.004	0.193	0.727	-0.920	1.305
	2	0.064	1.049	0.223	-0.680	2.778	0.172	-0.981	0.132	-2.278	0.316	0.165	-0.355	0.576	-1.636	0.926
	3	0.065	1.027	0.250	-0.770	2.825	0.175	-0.928	0.180	-2.313	0.458	0.175	-0.269	0.682	-1.597	1.059
	4	0.137	1.199	0.212	-0.734	3.133	0.217	-0.913	0.194	-2.323	0.498	0.247	0.002	0.997	-1.414	1.419
	5	0.275	1.514	0.161	-0.659	3.687	0.446	-0.850	0.214	-2.231	0.530	0.326	-0.326	0.658	-1.821	1.169
Total carotene (μg)	1	0.030	0.562	0.362	-0.681	1.806	0.001	0.073	0.897	-1.074	1.221	0.027	0.771	0.329	-0.811	2.352
	2	0.043	0.698	0.333	-0.758	2.155	0.104	-0.262	0.702	-1.650	1.127	0.176	0.648	0.395	-0.883	2.179
	3	0.048	0.699	0.341	-0.787	2.184	0.116	-0.127	0.862	-1.610	1.356	0.196	0.765	0.326	-0.797	2.327
	4	0.081	0.376	0.657	-1.357	2.110	0.168	-0.402	0.606	-1.991	1.186	0.292	1.013	0.190	-0.529	2.555
	5	0.226	0.842	0.392	-1.170	2.853	0.402	-0.141	0.858	-1.767	1.484	0.366	1.049	0.187	-0.542	2.640

**Supplementary Table S1.** *Cont.*																	
		**NAP**					**T2DM**					**NODAP**					
**Vitamin**	**Model**	**R^2**	**Unstandardised B**	***p***	**95% CI**		**R^2**	**Unstandardised B**	***p***	**95% CI**		**R^2**	**Unstandardised B**	***p***	**95% CI**		
					**Lower**	**Upper**				**Lower**	**Upper**				**Lower**	**Upper**	
Total retinol equivalents (µg)	1	0.028	0.624	0.373	-0.788	2.036	0.001	-0.154	0.845	-1.750	1.443	0.009	0.524	0.586	-1.412	2.461	
	2	0.040	0.783	0.354	-0.924	2.490	0.158	-1.279	0.180	-3.184	0.626	0.158	-0.167	0.877	-2.350	2.016	
	3	0.043	0.764	0.376	-0.985	2.513	0.160	-1.195	0.248	-3.274	0.884	0.170	0.042	0.970	-2.246	2.330	
	4	0.085	0.521	0.581	-1.410	2.451	0.215	-1.347	0.202	-3.467	0.772	0.266	1.028	0.405	-1.460	3.516	
	5	0.253	1.439	0.237	-1.026	3.904	0.430	-0.982	0.323	-3.004	1.040	0.330	0.790	0.550	-1.890	3.470	
Vitamin D (µg)	1	0.012	0.501	0.569	-1.283	2.286	0.011	-0.685	0.570	-3.122	1.751	0.045	0.896	0.205	-0.514	2.307	
	2	0.020	0.750	0.546	-1.772	3.273	0.150	-1.692	0.210	-4.396	1.013	0.169	0.632	0.505	-1.277	2.541	
	3	0.024	0.712	0.576	-1.879	3.303	0.164	-1.647	0.228	-4.390	1.097	0.182	0.623	0.515	-1.304	2.550	
	4	0.094	0.930	0.475	-1.720	3.579	0.219	-1.892	0.187	-4.765	0.982	0.252	0.400	0.676	-1.535	2.334	
	5	0.221	1.034	0.431	-1.655	3.724	0.472	-2.237	0.115	-5.071	0.597	0.321	-0.018	0.986	-2.050	2.014	
Vitamin E (mg)	1	0.000	-0.113	0.918	-2.327	2.102	0.018	0.769	0.459	-1.324	2.862	0.075	1.481	0.101	-0.303	3.265	
	2	0.007	-0.290	0.838	-3.182	2.601	0.106	-0.855	0.643	-4.600	2.890	0.157	-0.060	0.972	-3.530	3.409	
	3	0.015	-0.466	0.757	-3.535	2.604	0.120	-0.763	0.684	-4.566	3.041	0.171	0.265	0.882	-3.353	3.883	
	4	0.079	-0.621	0.686	-3.763	2.521	0.173	-1.253	0.518	-5.192	2.686	0.247	0.004	0.998	-3.664	3.671	
	5	0.195	0.225	0.896	-3.336	3.786	0.401	-0.200	0.926	-4.660	4.261	0.325	0.721	0.701	-3.092	4.534	

Abbreviations: HOMA-IR = homeostasis model assessment insulin resistance. NAP = Normoglycaemia after acute pancreatitis. NODAP = New-onset diabetes or prediabetes after acute pancreatitis. T2DM = Type 2 diabetes or prediabetes prior to acute pancreatitis. 95% CI = 95% confidence interval. Data are presented as R^2 values (from crude analysis), unstandardised B, *p* values (from linear regression) and 95% confidence intervals. All the variables were log-transformed. Model 1: unadjusted model. Model 2: age, sex, daily energy intake. Model 3: age, sex, daily energy intake, V/S fat volume ratio. Model 4: age, sex, daily energy intake, V/S fat volume ratio, alcohol intake, smoking status. Model 5: age, sex, daily energy intake, V/S fat volume ratio, alcohol intake, smoking status, aetiology of AP, number of AP episodes, cholecystectomy, use of antidiabetic medications. Significance was set at $p < 0.05$. Significant values are shown in bold.

Supplementary Table S2. Associations between habitual water-soluble vitamin intake and HOMA-IR in the study groups

Vitamin	Model	NAP					T2DM					NODAP				
		R^2	Unstandardised B	*p*	95% CI		R^2	Unstandardised B	*p*	95% CI		R^2	Unstandardised B	*p*	95% CI	
					Lower	Upper				Lower	Upper				Lower	Upper
Vitamin B1 (mg)	1	0.010	0.768	0.594	-2.149	3.684	0.001	-0.272	0.845	-3.086	2.541	0.102	2.426	0.054	-0.044	4.895
	2	0.027	1.658	0.471	-3.003	6.320	0.139	-2.053	0.273	-5.814	1.709	0.170	1.419	0.479	-2.618	5.455
	3	0.027	1.607	0.535	-3.660	6.873	0.148	-1.892	0.324	-5.764	1.979	0.182	1.344	0.507	-2.739	5.427
	4	0.083	1.384	0.617	-4.283	7.052	0.207	-2.346	0.235	-6.319	1.626	0.257	1.258	0.540	-2.890	5.407
	5	0.213	2.025	0.510	-4.290	8.340	0.412	-1.231	0.537	-5.321	2.860	0.325	0.862	0.687	-3.486	5.210
Vitamin B2 (mg)	1	0.038	1.503	0.304	-1.437	4.442	0.000	-0.007	0.996	-2.775	2.761	0.021	1.325	0.392	-1.776	4.426
	2	0.073	2.751	0.189	-1.442	6.944	0.129	-1.759	0.340	-5.474	1.957	0.168	-1.455	0.522	-6.030	3.120
	3	0.073	2.721	0.214	-1.677	7.119	0.141	-1.647	0.379	-5.434	2.139	0.179	-1.284	0.578	-5.940	3.372
	4	0.136	2.741	0.217	-1.731	7.213	0.182	-1.556	0.418	-5.449	2.338	0.256	1.578	0.577	-4.143	7.299
	5	0.245	2.566	0.274	-2.199	7.332	0.417	-1.510	0.466	-5.753	2.733	0.322	0.686	0.831	-5.835	7.207
Vitamin B3 (mg)	1	0.010	0.813	0.596	-2.290	3.917	0.004	-0.529	0.735	-3.691	2.634	0.069	1.760	0.116	-0.459	3.978
	2	0.024	1.438	0.501	-2.898	5.774	0.142	-2.426	0.252	-6.679	1.826	0.160	0.539	0.741	-2.752	3.830
	3	0.025	1.333	0.554	-3.257	5.924	0.156	-2.376	0.267	-6.684	1.933	0.174	0.627	0.703	-2.701	3.955
	4	0.100	1.868	0.419	-2.837	6.572	0.196	-2.464	0.298	-7.240	2.312	0.291	2.329	0.193	-1.245	5.904
	5	0.268	3.273	0.184	-1.692	8.237	0.432	-2.391	0.310	-7.175	2.393	0.361	2.268	0.210	-1.360	5.897
Vitamin B6 (mg)	1	0.020	1.227	0.455	-2.090	4.545	0.005	0.554	0.702	-2.376	3.484	0.023	1.207	0.368	-1.479	3.892
	2	0.053	2.929	0.274	-2.461	8.319	0.105	-1.026	0.661	-5.771	3.719	0.175	-1.556	0.410	-5.350	2.238
	3	0.053	2.897	0.309	-2.858	8.651	0.120	-0.947	0.689	-5.757	3.862	0.185	-1.408	0.463	-5.276	2.460
	4	0.109	2.720	0.350	-3.194	8.634	0.161	-0.640	0.796	-5.684	4.404	0.248	-0.267	0.896	-4.400	3.866
	5	0.242	3.301	0.288	-3.025	9.627	0.407	-1.169	0.651	-6.484	4.146	0.321	-0.118	0.954	-4.306	4.070

**Supplementary Table S2.** *Cont.*																	
**Vitamin**	**Model**	**NAP**					**T2DM**					**NODAP**					
		**R^2**	**Unstandardised B**	***p***	**95% CI**		**R^2**	**Unstandardised B**	***p***	**95% CI**		**R^2**	**Unstandardised B**	***p***	**95% CI**		
					**Lower**	**Upper**				**Lower**	**Upper**				**Lower**	**Upper**	
Vitamin B9 (μg)	1	0.018	0.765	0.474	-1.393	2.923	0.014	0.679	0.523	-1.469	2.828	0.034	1.242	0.277	-1.042	3.526	
	2	0.034	1.180	0.399	-1.654	4.013	0.100	-0.282	0.850	-3.319	2.755	0.157	-0.087	0.950	-2.913	2.738	
	3	0.037	1.150	0.421	-1.750	4.050	0.115	-0.241	0.873	-3.317	2.834	0.170	-0.041	0.977	-2.896	2.814	
	4	0.084	0.797	0.597	-2.282	3.876	0.161	-0.384	0.803	-3.531	2.763	0.261	1.134	0.466	-2.003	4.271	
	5	0.212	1.146	0.520	-2.514	4.807	0.406	-0.666	0.671	-3.891	2.560	0.331	1.010	0.530	-2.253	4.274	
Vitamin B12 (μg)	1	0.003	0.263	0.790	-1.744	2.270	0.031	-1.034	0.336	-3.195	1.128	0.010	0.568	0.549	-1.335	2.471	
	2	0.008	0.306	0.809	-2.272	2.884	0.196	-2.022	0.082	-4.319	0.274	0.163	-0.574	0.626	-2.948	1.800	
	3	0.012	0.231	0.860	-2.449	2.911	0.201	-1.939	0.105	-4.312	0.435	0.175	-0.466	0.698	-2.889	1.957	
	4	0.079	0.526	0.699	-2.255	3.307	0.232	-2.101	0.143	-4.963	0.762	0.248	0.104	0.934	-2.441	2.650	
	5	0.198	0.776	0.579	-2.091	3.642	0.439	-1.562	0.255	-4.341	1.217	0.321	-0.152	0.905	-2.757	2.452	
Vitamin C (mg)	1	0.028	0.648	0.375	-0.824	2.119	0.007	0.343	0.647	-1.171	1.857	0.046	1.080	0.203	-0.610	2.771	
	2	0.042	0.869	0.337	-0.957	2.694	0.099	0.052	0.952	-1.694	1.797	0.179	0.777	0.364	-0.943	2.496	
	3	0.044	0.841	0.366	-1.042	2.724	0.116	0.168	0.849	-1.629	1.966	0.194	0.803	0.352	-0.931	2.538	
	4	0.082	0.482	0.639	-1.621	2.585	0.162	-0.298	0.763	-2.313	1.718	0.261	0.620	0.471	-1.117	2.358	
	5	0.211	0.821	0.529	-1.863	3.506	0.404	-0.309	0.745	-2.262	1.644	0.329	0.518	0.583	-1.395	2.430	

Abbreviations: HOMA-IR = homeostasis model assessment insulin resistance. NAP = Normoglycaemia after acute pancreatitis. NODAP = New-onset diabetes or prediabetes after acute pancreatitis. T2DM = Type 2 diabetes or prediabetes prior to acute pancreatitis. 95% CI = 95% confidence interval. Data are presented as R^2 values (from crude analysis), unstandardised B, *p* values (from linear regression) and 95% confidence intervals. All the variables were log-transformed. Model 1: unadjusted model. Model 2: age, sex, daily energy intake. Model 3: age, sex, daily energy intake, V/S fat volume ratio. Model 4: age, sex, daily energy intake, V/S fat volume ratio, alcohol intake, smoking status. Model 5: age, sex, daily energy intake, V/S fat volume ratio, alcohol intake, smoking status, aetiology of AP, number of AP episodes, cholecystectomy, use of antidiabetic medications. Significance was set at $p < 0.05$. Significant values are shown in bold.
